# Gamma and beta bursts during working memory readout suggest roles in its volitional control

**DOI:** 10.1038/s41467-017-02791-8

**Published:** 2018-01-26

**Authors:** Mikael Lundqvist, Pawel Herman, Melissa R. Warden, Scott L. Brincat, Earl K. Miller

**Affiliations:** 1 The Picower Institute for Learning and Memory, Department of Brain and Cognitive Sciences, Massachusetts Institute of Technology, 43 Vassar Street, Cambridge, MA 02139 USA; 2 Computational Brain Science Lab, Department of Computational Science and Technology, KTH Royal Institute of Technology, Stockholm, 100 44 Sweden; 3 Department of Neurobiology and Behavior, Cornell University, Ithaca, NY 14853 USA

## Abstract

Working memory (WM) activity is not as stationary or sustained as previously thought. There are brief bursts of gamma (~50–120 Hz) and beta (~20–35 Hz) oscillations, the former linked to stimulus information in spiking. We examined these dynamics in relation to readout and control mechanisms of WM. Monkeys held sequences of two objects in WM to match to subsequent sequences. Changes in beta and gamma bursting suggested their distinct roles. In anticipation of having to use an object for the match decision, there was an increase in gamma and spiking information about that object and reduced beta bursting. This readout signal was only seen before relevant test objects, and was related to premotor activity. When the objects were no longer needed, beta increased and gamma decreased together with object spiking information. Deviations from these dynamics predicted behavioral errors. Thus, beta could regulate gamma and the information in WM.

## Abstract (web summary)

Previously, the authors have shown that working memory can be maintained by brief gamma oscillation bursts. Here, the authors use a new task to further demonstrate the dynamics of gamma and beta oscillations in working memory readout, independent of behavioral response.

## Introduction

Sustained spiking activity has been the dominant neural model of working memory (WM)^1–5^. The idea is that neurons, once activated by a stimulus, keep spiking, maintaining the representation of that stimulus. However, closer examinations of local field potentials (LFPs) that reflect coordinated population activity have revealed that complex dynamics underlie sustained LFP activity in the trial averages. In single trials, there are brief, discrete narrow-band oscillatory bursts in the gamma and beta bands^6^. The gamma bursts (~50–120 Hz) are tied to spiking carrying information about the remembered items. Beta bursts (~20–35 Hz) are associated with suppression of both informative spiking and gamma. These data are consistent with a model in which gamma-associated spiking stores memories by short-term changes in synaptic weights^7^. In this model, multiple items can be held in WM without mutual interference because gamma bursts active at different times store different items (time-division multiplexing).

It is unknown how working memory is controlled; how information is selectively encoded, read out, and forgotten when no longer needed. Here we investigated correlates of non-stationary dynamics in such control. Our model suggests that gamma should be correlated with spike information irrespective of the functional context, i.e., during encoding, maintenance, and readout. Gamma bursting should therefore increase and beta drop when WM is accessed. This is difficult to test in many experimental paradigms because readout often coincides with a behavioral response, hence adding a confounding or obscuring motor component. Thus, we turned to multiple-electrode data from a previously published experiment with a unique design^8, 9^. Monkeys determined whether a test sequence of two objects matched a sample sequence presented seconds earlier. They responded only after the full test sequence. Thus, we could examine WM readout and the animals’ evaluation of the first test object independent of motor activity.

As predicted, we found a ramping of gamma bursting in anticipation of WM readout. This was coupled with an increase in information specifically about the to-be-tested object and a decrease in beta at recording sites carrying information. Further, the use of a test sequence revealed that these readout dynamics only occurred when test objects were behaviorally relevant, suggesting volitional control. Consistent with this view, gamma and beta showed different dynamics for different types of match/non-match decisions (identity, order) and did so in a way that predicted different types of errors the animals could make. This lends support for the hypothesis that discrete oscillatory dynamics underlie maintenance, readout, and control of working memory.

## Results

### Task design

**Fig. 1:**
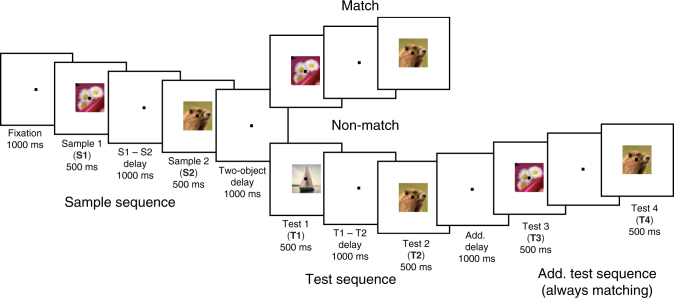
Experimental setup. The animals held a bar and fixated at the center of the screen throughout the task. Two sample objects (sample 1 and sample 2) were presented (500 ms), separated by a delay (1000 ms; S1–S2 delay). After another delay (1000 ms; two-object delay), a test sequence of two objects appeared (test 1 and test 2), separated by a 1000 ms delay (T1–T2 delay). The animals were trained to release the bar after the second test object only if both the identity and the order of the test objects matched the sample sequence. When the sample and test sequences did not match, the animals had to wait for the subsequent (always matching) test sequence to release the bar. Reused and modified with permission from^8^

On each trial (Fig. 1; Methods section), two sample objects were presented sequentially, separated by a 1 s delay. Then, after another delay, there was a sequence of two test objects, separated by a 1 s delay. If the identity and order of objects in the test sequence matched that of the sample sequence, animals were rewarded for releasing a bar. If the test sequence did not match, instead, monkeys had to maintain fixation and wait for a second, always matching test sequence. Upon the bar release following the second sequence, the monkeys received a juice reward (overall performance was 95.5%).

### Information encoding correlate with gamma not beta bursts

**Fig. 2:**
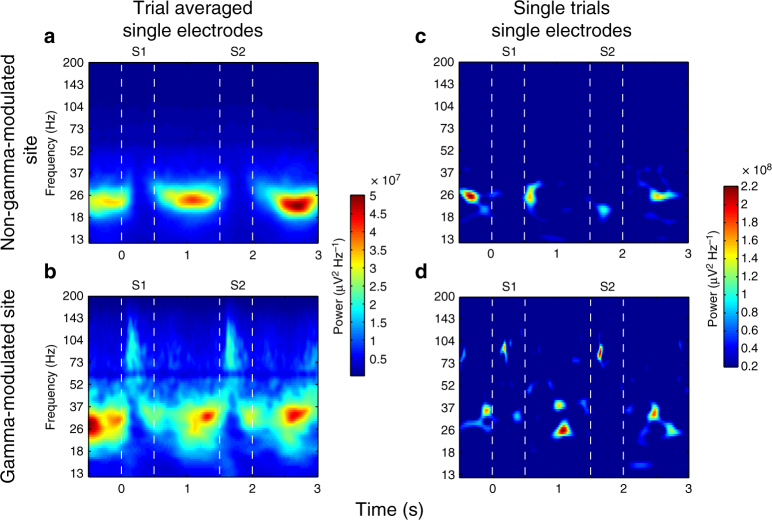
Trial-averaged and single-trial spectrograms. Example of trial-averaged spectrograms of a non-gamma-modulated **a** and a gamma-modulated **b** recording site. Displayed are the two sample object presentations, S1 and S2, and the following delays. Single-trial examples originating from the same two recording sites are shown in **c** (non-gamma-modulated) and **d** (gamma-modulated). Spectrograms were normalized by 1/f

**Fig. 3:**
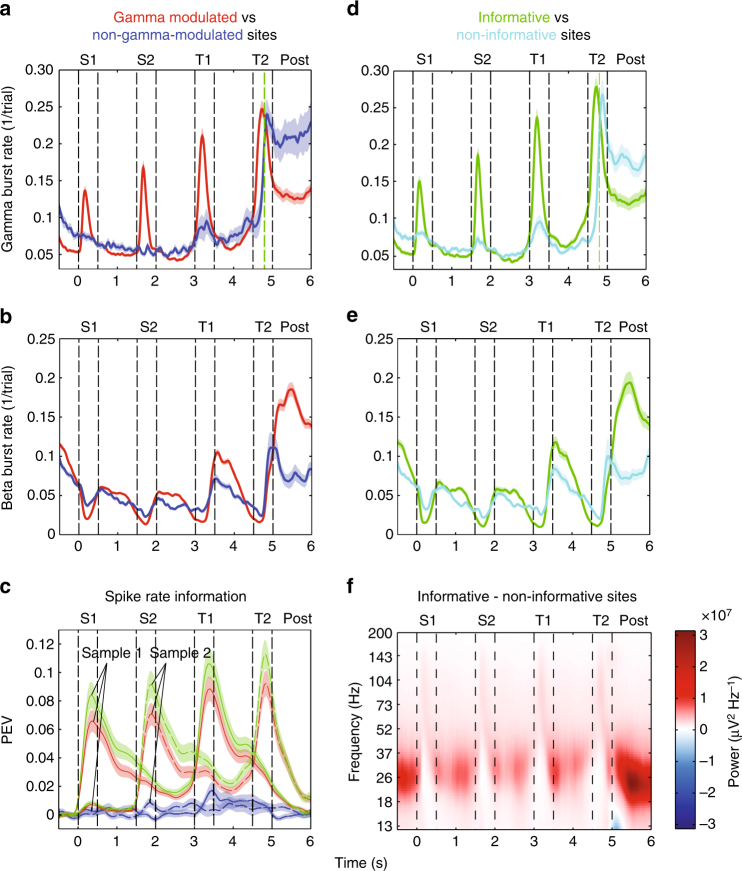
Burst rates and information. All plots show the mean estimates during correct trials with matching test sequences, and 1 s of post-trial (first 1000 ms after T2 offset on correct match trials, vertical green dotted line corresponds to the average time of response on these trials) activity. **a** Gamma burst rate for gamma-modulated (red, *n* = 160) and non-modulated (blue, *n* = 28) sites. **b** Same as in **a** but for beta burst rates. **c** PEV information about the identity of sample 1 (S1, solid lines) and sample 2 (S2, dotted lines) conveyed by firing rates in non-gamma-modulated (blue), gamma-modulated (red), and informative (sites containing at least one unit with significant PEV (Methods section), green) sites. **d** Same as in **a** but for informative (green, *n* = 130) and non-informative (light blue, *n* = 58) sites. **e** Same as **b** but for informative and non-informative sites. **f** Spectrogram showing average amplitude difference between informative and non-informative sites. T1 and T2 refer to the first and second test object, respectively. Error bars/shading correspond to SEM

As in prior work^6^, we found that LFPs, (*n* = 188 electrodes with at least one spiking neuron) recorded in prefrontal cortex (PFC; Supplementary Fig. 1) showed bursts of gamma and beta oscillations (Fig. 2). These gamma and beta oscillations were broadband and persistent over time in the trial-averaged data. However, as before^6^, on single trials there were actually brief narrow-band oscillatory bursts of varying central frequency (Fig. 2c, d; see also Supplementary Fig. 2 for summary statistics). The burst dynamics was highly variable across trials (Supplementary Fig. 3 showing gamma bursting for all trials of a single electrode), with brief high-power events that were only weakly correlated across trials (see Supplemental Text 1 and Supplementary Fig. 4 for an investigation of non-stationary nature of gamma). The rate of bursts (Fig. 3; Methods section) and their central frequency (Supplementary Fig. 2) were modulated over time, as seen in the trial averages.

Beta and gamma were anti-correlated over time (Figs. 2a–d, 3a–d). When gamma was high, beta was low, and vice versa. All recording sites showed beta oscillations that were elevated during fixation and in memory delays, and suppressed during object presentations (Fig. 3b). The majority of recording sites (160/188; *p* < 0.05) also exhibited increased gamma bursting when beta oscillations were suppressed (Fig. 3a, “gamma modulated” in red vs “non-gamma modulated in blue). Figure 3b illustrates the average beta burst rate at the gamma-modulated sites vs non-gamma-modulated sites. The beta modulation was significantly more pronounced at the gamma-modulated sites (red lines) than the non-gamma-modulated sites (blue lines). Beta bursting was lower during stimulus presentations and higher during delays at gamma-modulated relative non-modulated sites (Fig. 3b: *p* < 0.0001, S1–S2 delay: *p* = 0.005, S2: *p* < 0.0001, two-object delay: *p* = 0.011, two-sided permutation test). Thus, recording sites that had the strongest gamma modulation also showed the strongest beta modulation, but in opposite direction (across sites stimulus-induced (stim/pre-stim) beta and gamma were anti-correlated: rho = −0.57, *p* < 1e−16, Spearman’s rank correlation, *n* = 188; Supplementary Fig. 1, Supplementary Fig. 5c).

Modeling work predicted that gamma bursting (and suppression of beta) correlates with information in neuron spiking^7^. As in the previous study^6^, we first investigated whether gamma bursts were associated with spiking that carried information about the objects (informative spiking) at each site. For each isolated neuron, we measured information about the two sample objects by calculating the percentage of explained variance (PEV, Methods section) by object identity. Figure 3c shows the PEV averaged across all recording sites with at least one informative neuron (“informative sites”, 130/188). The PEV from gamma-modulated sites (red lines) was similar in strength and followed the same dynamic as that of the PEV from the informative sites (green lines). This was in contrast to spiking at non-gamma-modulated sites (blue lines) that carried virtually no information about the sample objects. The informative sites were strongly overlapping with the gamma-modulated sites (*p* < 6e−6, Fisher’s exact test for contingency between gamma-modulated and informative sites; Supplementary Fig. 1, Supplementary Fig. 5). Therefore, informative sites had very similar beta and gamma burst rates as gamma-modulated sites (Fig. 3d, e) with stronger burst rate modulation during samples and delays than on the non-informative sites (for PEV in burst rates, see Supplementary Fig. 6 and Supplementary Note).

While most recorded sites showed gamma modulation at sample onset, there was a wide distribution in modulation strength. Information in spiking (maximum PEV during the sample or delay period; for sample and delay independently see Supplementary Fig. 5) correlated positively with stimulus-induced gamma (Supplementary Fig. 5; rho = 0.49, *p* < 1e−16, Spearman’s rank correlation, *n* = 251. Informative neurons only: rho = 0.42, *p* = 2.5e−16, *n* = 187) and negatively with suppressed beta (rho = −0.44, *p* < 1e−16, Spearman’s rank correlation, *n* = 251. Informative neurons only: rho = −0.35, *p* = 4e−12, *n* = 187) during sample presentations. Thus informative sites tended to be the gamma-modulated sites with the strongest modulation of gamma (and suppression of beta). On the informative sites, gamma and beta bursting was anti-correlated over time (*r* = −0.40, *p* < 9e−14, *n* = 130, *t*-test with the null hypothesis for the mean *r* = 0 over sample and delays epochs combined), whereas on non-informative sites there was no correlation over time (*r* = 0.08, *p* = 0.12, *n* = 58). This is congruent with earlier findings suggesting that prefrontal beta and gamma are more modulated during task performance compared to passive fixation^10^.

In conclusion, as found previously^6^, there seemed to be a tight relationship between gamma modulation by sample object presentation and spiking that was informative about those objects. Since the gamma appeared in narrow-band bursts, it was unlikely to originate from the spectral contribution of spiking per se. In addition, there were no significant differences in average spiking rates between gamma-modulated and non-gamma-modulated sites (Supplementary Fig. 7). However, spiking at gamma-modulated sites showed a similar temporal profile as the gamma bursting, suggesting a mechanistic relationship.

### Gamma bursts correlate with information on single trials

We next investigated the relationship between bursting, spiking, and information on single trials (for details see Supplemental Text). During the first delay, spiking was elevated inside gamma bursts (*p* < 0.0001, *n* = 146, Wilcoxon rank test) and significantly suppressed during beta bursts (*p* = 0.004, *n* = 146; Wilcoxon rank test). Further, stimulus information in spiking (PEV) was significantly higher inside than outside gamma bursts (*p* = 0.02, *n* = 146, Wilcoxon rank test). The spike rate variance was elevated during gamma bursts (*p* < 0.0001, *n* = 146, Wilcoxon rank test) and reduced during beta bursts (*p* = 0.05, *n* = 146, Wilcoxon rank test) but not PEV (*p* = 0.14, *n* = 146, Wilcoxon rank test). These effects collectively suggested that gamma bursts corresponded to brief episodes of elevated spiking/information and beta bursts to reduced spiking. While the beta burst rate was elevated in delays and higher on informative than on non-informative sites (Fig. 3e, compare green and light blue lines: non-informative vs informative sites, S1–S2 delay: *p* = 0.001, two-object delay: *p* = 0.04, two-sided permutation tests), spiking information was correlated with gamma bursts also in delays. The temporal occurrence of gamma bursts on a particular site was only weakly correlated across trials, but gave rise to a unique trial-averaged temporal profile per site (Supplementary Note).

**Fig. 4:**
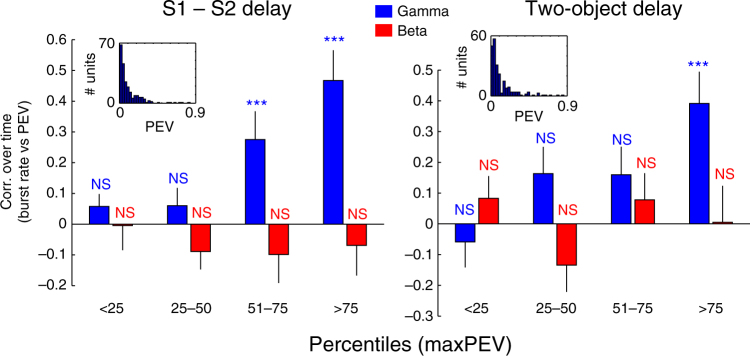
Temporal correlation of PEV information and burst rates. Correlation between temporal profiles of PEV of units with significant delay information (Methods section) and gamma (blue) and beta (red) burst rates recorded on the same site. The data are broken down into percentiles based on maximal PEV for each unit. In delay 1 (S1–S2 delay), burst rates were correlated with PEV information about the identity of sample 1 (left); in delay 2 (two-object delay), burst rates were correlated with the sum of PEV information about the identities of both samples 1 and 2 (right). *T*-test was used with *n* = 36 in each group. In delay, 1 only top two quantiles for gamma were significant (*p* = 0.005 and 3.2e−05, respectively); in delay 2, only the top quantile was significant (*p* = 0.001). Error bars correspond to SEM. Inlets show the distribution of peak PEV in each delay for all units with significant information (Methods section)

It has been observed that individual neurons carry information only transiently during delays^10–12^. We investigated whether the temporal profile of information in spiking correlated with the temporal profile of gamma bursting at the same recording sites, as suggested by our single-trial analysis above. This would explain how delay information could be associated with gamma even though beta bursting was on average elevated during delays. We found that information on a single-neuron level was positively correlated with gamma bursting across time (*r* = 0.23 in both delays, *p* < 0.00001, *t*-test, including neurons with significant delay information, *n* = 146) and uncorrelated with beta bursting (*r* = −0.07 in first delay, *p* = 0.06, *r* = 0.01 in second delay, *p* = 0.73, *n* = 146) during delays. Within the population of informative neurons, most had low PEV values, while a small population was highly informative (Fig. 4). The correlation between gamma burst rates and information over time during delays was driven by this group of highly informative neurons that tended to be strongly correlated with gamma (Fig. 4). In addition, firing rates of the delay-selective neurons were correlated with gamma (*r* = 0.32, *p* < 1e−13, *n* = 146) and weakly anti-correlated with beta (*r* = −0.10, *p* = 0.05, *n* = 146).

### Post-trial beta bursting as a mechanism for WM reallocation

We analyzed the post-trial period when the animals were no longer restricted to maintain central fixation. We found elevated rates of both beta and gamma bursting (Fig. 3a, b, d, e). This elevated bursting could have been generated by motor and sensory events. However, a comparison of post-trial burst rates between informative and non-informative sites yielded significant differences, which could not be explained by such events. Post-trial gamma bursting (Fig. 3a, d) was now higher in the non-informative compared to the informative sites (*p* < 0.0001, two-sided permutation test). In contrast, post-trial epoch beta bursting in informative sites was high (Fig. 3e, between 5 and 6 s), whereas beta in non-informative sites was relatively suppressed (*p* < 0.0001, two-sided permutation test). In fact, the most pronounced difference between informative and non-informative sites at any time and frequency was this elevation of beta bursting in informative sites after the end of each trial (Fig. 3f, Supplementary Fig. 7b). During this time, information about the objects (which was no longer needed) dropped (compare dotted green line at 3 and 6 s in Fig. 3c. Average T2/S2 PEV in the first 1 s following T2 offset compared to average S2 PEV following S2 offset, *p* < 0.0001, *n* = 146, two-sided permutation test).

### Gamma ramp-up reflects information read out

We next investigated readout from WM. There was a ramp-up of gamma bursting just before presentation of the first (T1; *p* < 0.0001, Fig. 3a, d) and second test object (T2, *p* < 0.0001, informative sites, *n* = 130, two-sided permutation test on informative sites, *n* = 130. For all comparisons here, ramp-up was evaluated comparing statistics between two 300 ms epochs separated by 100 ms to avoid smoothing between the following intervals: 300–600 ms and 700–1000 ms of the delay). The ramp-up was especially pronounced for gamma on informative sites (Fig. 3d, green line).

**Fig. 5:**
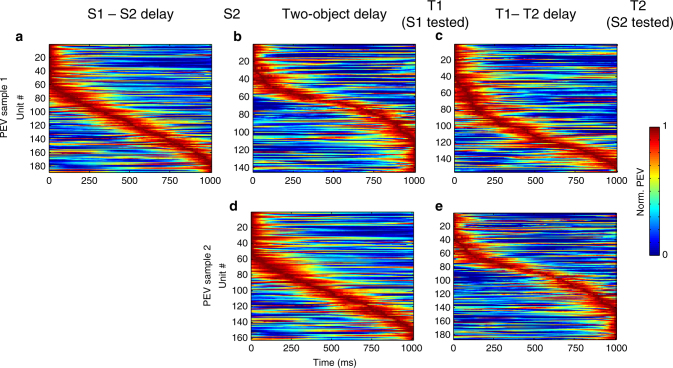
PEV information during different types of delays. The data from trials with matching test sequences. Plotted is the normalized PEV information about sample 1 (S1; top panels: **a**-**c**) or sample 2 (S2; bottom panels: **d**, **e**) during S1–S2 delay (left column), two-object delay (middle column) and T1–T2 delay (right column) for all units with significant PEV (*p* < 0.01; ANOVA, tested in each delay and for each sample independently). Units are sorted based on the timing of peak PEV in each delay. Following two-object delay, S1 is tested. Following T1–T2 delay, S2 is tested. Included in each plot are all neurons that had significant PEV in that particular delay (*n* = 144–188)

Objects were tested one by one, in a sequence. Information only ramped up for the sample item that was about to be tested, i.e., information about the identity of the first sample object ramped up before the first test object, and information about the second sample object ramped up before the second test object (Fig. 3c; Supplementary Fig. 8, PEV about S1 before T1, *p* = 0.003. PEV about S2 before T2, *p* = 0.0005, permutation test, *n* = 188). For objects in the sequence that were not relevant for the upcoming test, there was instead a non-significant decreasing trend (PEV about S2 before T1, *p* = 0.37; PEV about S1 before T2, *p* = 0.29, permutation test, *n* = 188). To investigate this further, we sorted neurons by when in the delays they carried information (Fig. 5). If a sample was not the one being tested next (e.g., the second sample object in the delay before the first test object), neural information about that object was evenly spread over the delay (Fig. 5a, c, d). For objects about to be tested, instead, selective neurons tended to show a peak in PEV just before the test (Fig. 5b, e), creating a ramp-up on the population level. Correspondingly, there was a strong correlation between gamma and spike rate ramp-up (T1: rho = 0.383, *p* < 1e−5; T2: rho = 0.441, *p* < 1e−7, *n* = 146, Spearman’s rank correlation), without a significant correlation between gamma and PEV ramp-up on a per site basis (T1: rho = 0.053, *p* = 0.378, *n* = 35; T2: rho = 0.123, *p* = 0.178, *n* = 37).

We interpreted the ramp-up of gamma bursting as readout of WM in anticipation of the decision about matching vs non-matching test stimuli. The ramp-up did not account for prediction of presentation of any object, only the test objects. There was no ramping of gamma bursts (rather a non-significant decrease prior to S2; *p* = 0.10, *n* = 130) or spiking PEV before presentation of the first or second sample objects (Fig. 3c, d), even though both events were predictable. In trials in which the first test sequence did not match, it was immediately followed by a second, matching test sequence (Fig. 1). There was no gamma burst rate (T3; *p* = 0.46, T4; *p* = 0.23, *n* = 130) or spiking PEV increase prior to these test objects (T3; *p* = 0.17, T4; *p* = 0.27, *n* = 188). Thus, there was a ramp-up before T1 (which had to be evaluated but never responded to) but not before T4 (which did not need to be evaluated but always responded to). This associated the ramp-up with readout of sample information and dissociated it from motor response. Finally, the motor response was always the same (a bar release). Thus, motor activity could not explain that information selectively about the to-be-tested object increased.

In conclusion, gamma and beta bursting was in a push–pull relationship, where gamma was associated with informative spiking. Next, we investigated this dynamics following matching and non-matching test objects.

### Gamma and beta react differently to matches and non-matches

The task required matching a sequence of two test objects to a sequence of two sample objects. This allowed us to examine neural activity associated with different types of non-matching object/stimuli configurations (object order vs identity). We focused on the first test object, T1, and the following delay because there was no behavioral response during these epochs.

**Fig. 6:**
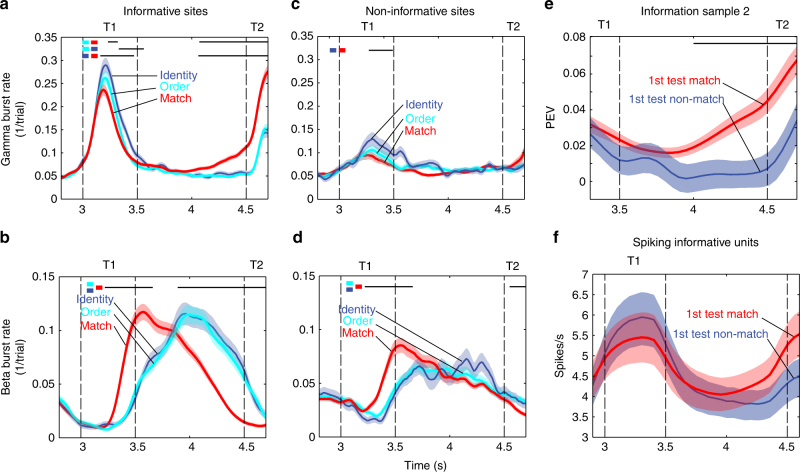
Burst activity around the first test. Plots show burst rates during presentation of the first test object (T1) and the following delay for correct trials. **a** Gamma burst rate for matching (red) and non-matching sequences (object identity violation, dark blue; order violation, turquoise) in sites with at least one unit carrying significant information about the identity of sample 1 or 2 (*n* = 130). Black horizontal lines denote intervals with significant differences between burst rates in conditions indicated by the corresponding colored squares (*p* < 0.05; cluster-based statistics with permutation test). **b** Same as **a** but for beta burst rate. **c** Same as **a** but for sites with no informative units (*n* = 58). **d** Same as **b** but for sites with no informative units. **e** PEV information about the identity of sample 2 (tested after the delay at T2) for units with significant PEV/information during delay (using neurons that also had LFPs recorded; *n* = 146) in two groups of trials: with matching (red) vs non-matching (blue) pair of objects, S1 and T1 (at first test). Black horizontal line marks an interval where the PEV means in the two groups of trials are significantly different (*p* = 0.001; one-sided cluster-based permutation test). **f** Average firing rates at informative sites (*n* = 189) for first test matching (red) and non-matching (blue) conditions (analogously to **e**)). Error bars/shading correspond to SEM

When the first test object did not match either of the sample objects, it was termed an “object non-match”. When the first test object matched the second sample object, it was referred to as an “order non-match”. We found that gamma bursting during test object presentation distinguished between a match and different types of non-matches (Fig. 6a). During presentation of the first test object, the gamma burst rate was lowest for a match, highest for an object non-match, and intermediate for an order non-match case (horizontal black lines in Fig. 6a denote intervals when burst rates for object non-match and match were significantly different, *p* < 0.05; cluster-based statistics).

By contrast, the timing of the beta bursts in the delay following the first test object was distinctive for matching and non-matching configurations (Fig. 6b, black line denotes significant differences between match relative both object non-match conditions, *p* < 0.05; cluster-based statistics). However, it did not distinguish between different types of non-matches. There was no significant difference between order and identity non-matches. The differences in gamma bursting between conditions were short-lived and disappeared shortly after test object presentation. The differences in beta bursts had longer duration, and bridged the 1-s delay to the second test object. Around T1, there was spiking and bursting information about the samples, the test object and its match status (Supplementary Fig. 9).

### Gamma ramp-up seems to be under volitional control

Gamma burst rate also ramped up as the presentation of the second test object approached, but only if the first test object was a match (and thus further readout was needed, Fig. 6a, red line). If the first test object was either type of non-match (blue and cyan lines), there was no gamma ramp-up (Fig. 6a). As the monkeys had to keep fixation also for T2 regardless of whether T1 was a match or not, this difference could not be explained by saccades. Presumably the animal could have already made its non-match decision after the first test object did not match. Indeed, the corresponding ramp-up of information in spiking about the second sample occurred only if the first test object was a match (Fig. 6e; *p* = 0.001, cluster-based statistics with permutation test, *n* = 146). All these effects were stronger at the informative sites (Fig. 6a, b) than at the non-informative sites (Fig. 6c, d). The gamma ramp-up before the second test object in match trials and the corresponding increase in beta bursting in non-match trials were only seen on informative sites. Non-informative sites showed no significant differences (Fig. 6c, d). Spike rates on informative sites displayed similar tendencies as gamma bursting, but with no significant differences between match and non-match conditions (Fig. 6f).

### Gamma and beta reflects different types of errors

We examined trials in which the monkeys made errors in match/non-match judgments (i.e., incorrect behavioral response after the second test object). For this analysis, we focused on informative sites because they showed the most robust effects (see above). We combined both types of non-match trials (object and order) to obtain enough incorrect trials for statistical analysis.

**Fig. 7:**
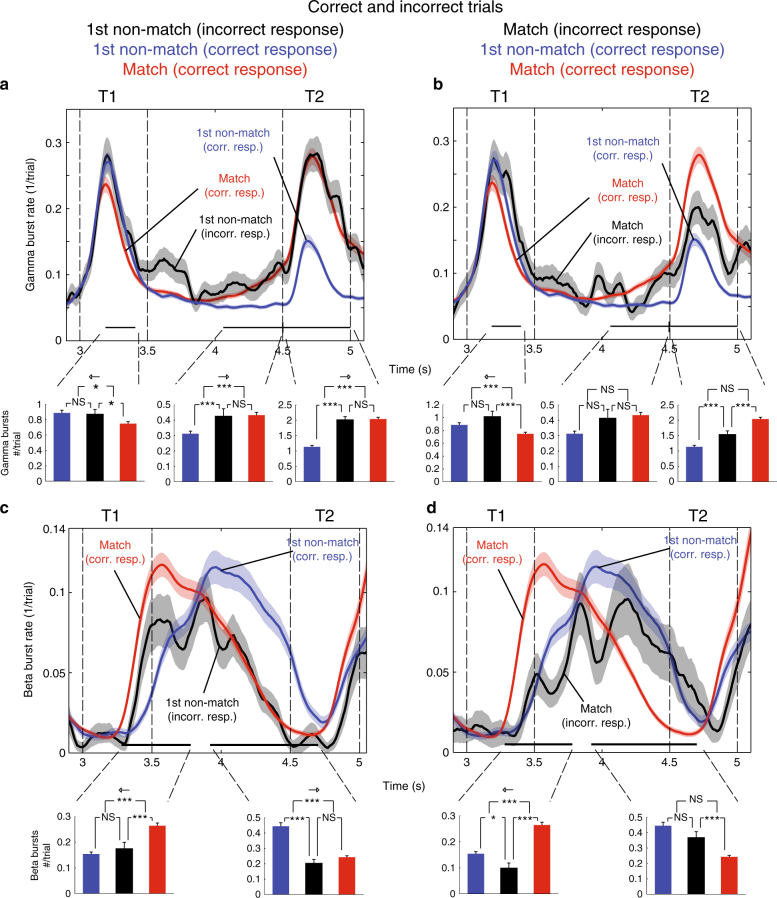
Bursts dynamics for incorrect trials. Bursting dynamics during and following the presentation of the first test object, T1, on informative sites (*n* = 130). The corresponding burst rates for the correct matching (red) and correct first test non-matching, i.e., T1≠S1 (blue) conditions are given as reference. Black curves illustrate burst rates for incorrect trials in the matching (right panel column) and first test non-matching (left panel column) conditions. Black horizontal lines at the bottom of each panel denote intervals when the burst rates in the correct matching and correct non-matching conditions are significantly different (*p* < 0.05; cluster-based permutation test). The bar plots below represent the average number of bursts per trial and recording site for the three conditions in the corresponding panel within the aforementioned intervals. Planned pair-wise comparisons between these mean statistics (the incorrect condition was compared to the two correct conditions) were conducted using two-sided permutation tests. In addition, we tested the hypothesis that burst counts on incorrect trials were equidistant (Methods section) to the two correct conditions (arrow indicate which condition the error trials were more similar to for significant effects). **a**, **b** Show gamma burst rates, and **c**, **d** show beta burst rates. For correct trials: *n* = 130 (number of electrodes), whereas for the incorrect matching trials: *n* = 117 (as some sessions were devoid of error trials of this kind) and for the incorrect non-matching trials: *n* = 118. Error bars/shading correspond to SEM

Figure 7 shows the average gamma and beta burst rates on error trials to correct trials. First, we considered errors when the first test object did not match the corresponding first sample (Fig. 7a, c). During the first test object, the gamma burst rate on these trials (black curve) overlapped with the gamma burst rate from correct non-match trials (blue curve). The number of gamma bursts was significantly different from that in correct match trials but not from that in correct non-match trials (Fig. 7a, bottom) during the first test. The same was true for beta bursting (Fig. 7c). Thus, it seemed that in trials in which the monkeys mistakenly responded “match” to a non-matching sequence, the gamma and beta burst rates during the presentation of the first, non-matching test object followed the “correct” trajectory (i.e., as if the first test object was a non-match). Instead, the error seemed to arise in the delay after the first test object. Interestingly, in that delay period there was a ramp-up of gamma burst rate in non-match trials with incorrect responses (black line), which closely followed the gamma burst rate on match trials correctly executed by the animals (red line). During the last part of the delay leading up to the second test object, the average number of gamma bursts in incorrect non-matching trials (Fig. 7a, bottom, black bar) was not significantly different from correct match trials (red bar), but it was significantly different from that in correct non-match trials. This discrepancy between correct and incorrect trials was reflected also in beta bursting. The second half of the delay beta was suppressed in incorrect relative correct non-match trials, but not significantly different from correct match trials (Fig. 7c, bottom). In sum, when the first test stimulus was a non-match, the gamma and beta burst rates followed the average trajectory observed for correct identification. The error in responding “match” seemed to occur in the second half of the delay as the gamma and beta burst rates became more similar to the profiles of match trials.

Next we examined trials with matching test sequences, where the monkey failed to respond, as if the sequence did not match (Fig. 7b, d). In this case, the gamma burst rate in response to the first (matching) test object (black line) virtually overlapped with that in correct trials when the first test object was a non-match (blue line). The number of gamma bursts during the first test object (black bar) was not significantly different from that in correct non-match trials (blue bar), even though the first test object was actually a match (Fig. 7b). The number of beta bursts was likewise significantly lower than that in correct match trials (Fig. 7d). In the second half of the delay, leading up to the second test, beta bursting was not statistically different from that in correct non-match trials and significantly higher than that in correct match trials. There was no significant difference in gamma bursting between the incorrect (black line and bar) and correct match (red line, bar) nor correct non-match trials (blue line, bar). During the presentation of the second test object, however, the error gamma burst rate was between, and significantly different from gamma burst rates corresponding to both correct match (red bar) and correct non-match trials (blue bar).

Finally, we wanted to rule out the possibility that differences in burst rates between conditions were due to shifts in baseline power. For all intervals, shown in Fig. 7, where we tested for significant differences in burst number between the conditions (correct match, incorrect match and both sets of error trials), we also tested for differences in average power during bursts (Supplementary Table 1; Methods section). There were no significant differences in power during beta bursts between any conditions. For gamma there were only significant differences at the second test, between conditions where the animals produced a motor response in one condition but not the other. This demonstrated that changes in burst rates were not the result of thresholding of signals with different, tonic means. Instead, it suggested that the behavioral correlates of matching and non-matching test objects differed in the rate of burst occurrences.

## Discussion

We analyzed LFPs and spiking activity during a sequence working memory task^8, 9^. The task structure gave us insights into neural control over working memory, as monkeys readout an object sequence from working memory and compare it to a sequence of test objects. The analysis was driven by predictions from a working memory model^7^. We found brief gamma and beta bursts that seemed to have different functions, confirming and extending previous results^6^. Gamma bursts were temporally and spatially linked with the expression of sensory information in spiking during encoding and delays. The observed interaction between timing of bursts and information on single-trials implies that bursts did not reflect noise fluctuations. Beta bursts were associated with suppression of gamma and suppression of object information in spiking. Gamma and beta bursting was anti-correlated over time, but only at recording sites where spiking carried information about the to-be-remembered objects (informative sites). The beta and gamma interplay suggests a potential mechanism for controlling working memory^6, 7^. The balance between beta and gamma would control the level of gamma bursting and hence, the expression of sensory information in spiking linked to gamma. The results suggest that beta/gamma balance is under volitional control. The balance was, along with information, flexibly modulated by task demands absent of sensory stimuli.

This was reflected in beta and gamma activity during working memory readout and match/non-match decisions. On sites that had information in spiking, the rate of gamma bursts ramped up, and beta rates decreased, at the end of memory delay in anticipation of the comparison of the memories to the forthcoming test objects. This was accompanied by an increase in end-of-delay spiking that is often seen in WM tasks^11–13^. Here we observed it in the absence of, and unrelated to, any forthcoming motor response, as did Hussar and Pasternak^12^. Further, we found the ramp-up in spiking carried the specific object information needed for the immediately forthcoming decision (e.g., first sample object information for comparison to the first test object, and so on). The gamma/informative spiking ramp-up did not occur in anticipation of just any expected event. Sample object presentation was also predictable, but no ramp-up was present. Thus, gamma ramp-up coincides with working memory readout, not anticipation of object presentations. Importantly, it did not occur before the second test object, if the first test object was a non-match. This rendered the whole sequence a non-match and the second test object was no longer relevant. On these trials, there was an increase in beta at the sites normally carrying spiking information. Thus, the gamma and informative spiking ramp-up was regulated depending on behavioral relevance.

These dynamics continued to play out in the comparison between the memories and the test objects. Gamma bursting was highest for identity non-matches, second highest for order non-matches and lowest for matches. This reflects their relative level of “non-matching”. It mirrors observations of changes in average spike rate in prefrontal cortex^4, 14^. The changes in gamma were then followed by changes in beta. When the first test was a match, beta was elevated immediately after its offset. When it was a non-match, the beta increase came later, just before the second test object, as if preventing readout of the now irrelevant second object. Similarly, it has been reported that prefrontal sites showing beta to matching test stimuli respond with a shorter latency than sites preferring non-matching test stimuli^10^.

Deviations from the match and non-match gamma/beta dynamics predicted behavioral errors. When the first test object was a non-match, initial gamma bursting reflected its non-match status, whether or not animals made an error. The “match error” instead crept into both gamma and beta bursting later, in the delay between the two test objects. Then gamma and beta bursting reached levels similar to when animals correctly identified a match. There was a gamma increase and a beta suppression. It was as if animals expected to make decision about the second test object. However, that was only necessary if they thought the first test was a match. When, instead, the first test object was a match and animals subsequently responded non-match, the error was instead immediate. The gamma (and subsequently) beta burst rate induced by that object was similar to non-matching, rather than matching, test objects.

These dynamics were largely confined to sites in which the spiking contained working memory information. The most striking difference between informative and non-informative sites was however after the behavioral response, in the post-trial epoch. At informative sites, beta was particularly high and gamma low. At this point, the memory content is no longer relevant. Thus, the shift to activity dominated by beta may clear out working memory in preparation for the next trial by suppressing gamma. Indeed, at the same time, spiking information about the last object held in working memory (the second sample/test object) decreased dramatically, as if suppressed. Thus, taken together, the model and our findings suggest cortical beta as a spatiotemporal filter, dictating when and where sensory information is encoded and retained. It has been suggested that alpha oscillations (8–14 Hz) have similar inhibitory role in sensory-motor areas in delay match to sample tasks^15^. In general, sensory alpha has been suggested as having inhibitory functions^16^, and it might be that beta has a similar role but that the frequency is shifted upward in higher-order cortex. Beta oscillations are likely produced by the interactions between mediodorsal thalamus and prefrontal cortex^17, 18^. Thus, we hypothesize that this network might be involved in regulating working memory activity^18^, while superficial layers of prefrontal cortex may contain the contents itself^7^.

Cortical gamma has long been seen as a correlate of sensory processing^19^ but the role of beta has been more elusive^20–25^. Beta has been suggested as an inhibitory rhythm^15, 20, 21^, to be involved in motor maintenance^22^, post-movement rebound^23, 24^ or a mechanism to preserve status quo^25^. The interplay between oscillations and spiking observed here seems congruent with an inhibitory role of beta. Increases in beta were correlated with suppression of gamma and informative spiking. Beta was also elevated post-trial on informative sites, when information needed to be cleared out. This interpretation could explain why motor beta is most pronounced after a completed movement^23, 24^, when the movement plan should be forgotten. Further, in the human ventral stream, different patterns of gamma were induced by different visual stimulus categories, while beta was globally reduced^26^. It is therefore possible that higher-order cortex added volitional control onto mechanisms similar to those found in visual cortex. Recent findings suggest that sensory beta may be modulated by prefrontal cortex during spatial attention, and anti-correlated with subsequent gamma evoked by the target^27^. The beta observed in this study was in the high-beta (β2) range, and beta oscillations in the β1 frequency band might have different behavioral correlates^28–30^.

Sustained spiking has often been seen as the neural correlate of working memory^1–4^. It has been modeled by attractor networks with persistent activity^5^. The activity in such networks is by definition stable to perturbations. Here the observed dynamics was not sustained, but occurred in brief bursts. Dynamically speaking, brief bouts of gamma and informative spiking, with interleaved periods of silence might be a way to combine the robustness of attractor-like activity with more flexible computations^31–34^. If gamma bursts correspond to periods of short-lived attractors^7^, the periods of silence between them might be opportunities for the network to evolve and weave in new information. Time-varying signals in working memory delay activity appears to be a hallmark of prefrontal dynamics^4, 9, 34–39^. We suggest that fast transitions between brief high-power events in gamma and beta allow for the flexible coordination of multiple items held in working memory.

## Methods

### Behavioral task and data collection

We re-analyzed data from two previous studies^8, 9^. For details of data collection and task structure, we refer to these studies, but in short: the task was structured such that the animals had to compare encoded objects sequentially to test stimuli. Each trial (Fig. 1) consisted of an encoding phase in which two objects (out of four possible each session) were presented sequentially, separated by a 1 s delay. The second sample object was followed by another 1 s delay and then a sequential test phase (also containing two of the four possible objects). The identity, as well as the order of the items in the test sequence, had to match that of the to-be-remembered sequence for the correct response to be ‘match’. The monkeys reported a matching test sequence by releasing a bar. If the first test sequence did not match, the monkeys had to wait for the second test sequence (always matching) before releasing the bar and receiving a juice reward (overall performance was 95.5% correct). This second matching test sequence was to ensure that the animals were engaged also in the non-match trials and to remove false positives. Throughout the whole trial, including test sequences the animals had to fixate on a dot in the center of the screen and all items were presented in the same location, at the fixation dot (Fig. 1). Thus, item information was not confounded with location information and planned saccades at any part of the task.

The data were recorded from two adult rhesus monkeys (Macaca mulatta; monkey S, female, and monkey A, male). The animals received postoperative antibiotics and analgesics and were always handled in accord with the National Institutes of Health guidelines, and all procedures were approved by the Massachusetts Institute of Technology Committee on Animal Care. For each recording, a new set of acute electrodes (up to eight simultaneously) were lowered through a grid. The PFC was randomly sampled without any pre-screening for informative neurons and all isolatable neurons were kept. Supplementary Figure 1 shows the distribution of anatomical recording sites in the two monkeys. The LFPs were recorded at a sampling rate of 1 kHz. For details please see Warden and Miller^8^, as well as Warden and Miller^9^, in which the original data were recorded. We used all the data of these two studies in which the animals performed the recognition task. In one data set, Warden and Miller^8^, LFPs were not always recorded with the spikes. For analysis in which we analyzed spike-field interactions, only neurons with a simultaneously recorded LFP were included. In a smaller subset of analysis (Fig. 5; Supplementary Fig. 8), we also included neurons missing LFP recordings. All available LFPs (without artifacts) from both data sets were always included.

### Signal processing

At first, all electrodes without any isolatable neurons were removed. Then, a notch filter with constant phase across a session was applied to remove 60-Hz line noise and its second harmonic. Two methods for the LFP spectral estimation were employed: Morlet wavelet analysis^40^ and multi-taper approach with a family of orthogonal tapers produced by Slepian functions^41, 42^. They yielded very similar results in terms of qualitative time-frequency content. They also led to comparable burst extraction outcomes. For all the presented spectrograms (except Fig. 2 where wavelets were used) and for burst analysis the multi-taper approach was adopted with frequency-dependent window lengths corresponding to four to eight oscillatory cycles and frequency smoothing corresponding to 0.2–0.3 of the central frequency, *f*_0_, i.e., *f*_0_ ± 0.2*f*_0_, where *f*_0_ were sampled with the resolution of 1 Hz (this configuration implies that one to three tapers were used). The spectrograms were estimated with the temporal resolution of 1 ms. On some sessions there were high-power, broadband frequency artifacts; these sessions were discarded from further analysis.

### Burst extraction and detailed estimation burst attributes

The bursts were calculated similarly as in the previous study^6^ with the only difference in estimating the reference mean and standard deviation of spectral band power. Here, the statistics were obtained over the 10-trial-long period (the last nine plus the current trial) to minimize the potential effects of removing true effects in trial to trial differences in power between conditions.

The first step of the oscillatory burst identification consisted in extracting a temporal profile of the LFP spectral content within a frequency band of interest. We used single-trial spectrograms, obtained with multi-taper approach, to calculate smooth estimate of time-varying band power. Oscillatory bursts were recognized as epochs during individual trials when the respective measure of instantaneous spectral power exceeded the threshold set as two SDs above the mean of the respective band power over the 10-trial-long reference period, providing that they lasted at least three oscillatory cycles (for the mean frequency of the band of interest). To obtain a more accurate estimate of burst duration, the time-frequency representation of the signal was extracted in the spectro-temporal neighborhood of each burst using the multi-taper method with the aforementioned smoothing configuration, and two-dimensional Gaussian function was fitted to the resulting local time-frequency map. The burst length was then defined as a time subinterval where the band average instantaneous power was higher than half of the local maximum (half-power point) estimated using the Gaussian fit. The frequency coordinate of the peak of the Gaussian fit was recognized as the central burst frequency and the burst’s frequency width was defined analogously to the burst length but in the frequency domain. For each, burst the spectro-temporal power average was calculated and normalized with reference to the session power spectral average within a narrow band around the central frequency of a given burst.

Finally, based on burst intervals extracted from each trial for the beta band (20–35 Hz) and two gamma sub-band oscillations (50–90 and 80–120 Hz), we defined for each band a trial-collective measure, called a burst rate, as the proportion of trials where a given electrode displayed burst-like oscillatory dynamics around the time point of interest sliding over the trial length. In other words, a burst rate corresponds to the time-varying likelihood of a burst occurrence on a given electrode at a specific time point in the trial (1/trial). Burst rates were estimated for beta and gamma sub-bands. For all figures and statistics involving gamma burst rates we used the summed burst rates of the two sub-bands. Based on the estimated central frequency (above) each burst was exclusively assigned to one of the two gamma bands in the case of bursts spanning both sub-bands.

### Selection of informative cells/sites

The instantaneous firing rates were estimated for each neuron by convolving spike trains with a Gaussian kernel (50 ms total width). As a control for the analysis in Fig. 4, we also used spike kernels of 80 and 120 ms to match the smoothing in gamma and beta power estimates, respectively. This yielded quantitatively very similar correlation results as the 50 ms spike kernel. The bias-corrected PEV^43^, *ω*^2^, was then estimated from firing rates with the resolution of 1 ms across trials with different stimulus dependent conditions. As a result, PEV allowed for the quantification of information associated with the modulation of firing rates (variance) of individual neurons depending on the stimulus condition. This way, for example, we could estimate the amount of variance-based information carried by individual neurons about the identity of the presented object. The bias-correction minimizes the problem of non-zero mean PEV for small sample sizes.

$$\omega ^2 = \frac{{{\mathrm{SS}}_{{\rm Between}\,{\rm groups}} - {\mathrm{df}} \times {\mathrm{MSE}}}}{{{\rm SS}_{{\rm Total}} + {\rm MSE}}},$$

where MSE is the mean squared error, df the degrees of freedom, SS_Total_ the total variance (across all trials), and SS_Between groups_ the variance between groups of trials formed w.r.t. stimulus condition of interest. We used one-way and multi-way ANOVA for the condition of interest to recognize informative neurons, with very similar results. A neuron was defined as informative if the ANOVA analysis provided statistically significant evidence (*p* < 0.05, Bonferroni corrected for testing at multiple time points) for the rejection of null hypothesis, thus yielding relevant group-dependent effects (*ω*^2^) at any time point during presentation and delay periods. PEV information was also calculated based on the gamma and beta burst activity using the same bias-corrected approach as for firing rates. The feature used for estimating oscillatory burst PEV was the proportion of time within a moving window of analysis that was occupied by a burst, estimated in each trial. Burst PEV estimates were robust to the length of the analysis window ranging from 50 to 150 ms. In the end, the windows size was fixed to 100 ms.

### Statistical methods

The majority of tests performed (all burst rate comparisons and PEV comparisons) in this study were non-parametric due to insufficient evidence for model data distributions. To address multiple comparisons problem, we employed permutation, Friedman’s and Wilcoxon’s signed-rank tests where appropriate. We also performed Pearson’s rank correlation or Student’s *t*-test for non-zero mean for correlations between burst rates and PEVs. For details see below.

### Correlation

We also estimated the correlations between the measures of time-varying spectral band content, burst rate statistics and PEV profiles over time in WM delays. These measures are by definition estimated over a set of trials (collective measures) and we used trial averaged signals on individual recording sites. For example, gamma burst rate was correlated with the beta burst rate and spike PEV over time, on the same electrode.

In addition, we correlated information with induced gamma and beta bursting. To this end, we calculated the average burst rate across all presentations (500 ms) divided by the average of all preceding epochs of fixation (500 ms). For PEV information, we estimated the maximum PEV value during the presentation and the following delay. For each neuron, we thus obtained one data point. Next, we correlated the resulting data points across the population. To mitigate the biased effect of non-uniform distribution of PEVs (a large number of close-to-zero values and a low number of high values), we resorted to Spearman’s rank correlation.

Finally, some attention should be given to the way we report correlations between the measures of time-varying spectral band content, burst rate statistics and PEV profiles. The correlation analyses were performed on individual electrodes and only the summary statistics (mean and SEM) were presented.

### Error trial analysis

In order to investigate whether incorrect trials exhibited similar burst characteristics as in matching or non-matching correct trials in different epochs during the test period (T1, T2, and the delay between T1 and T2), we performed the following analysis. First, we defined intervals of potential interest based on the statistical comparison of temporal profiles of burst rates in correct non-matching vs correct matching trials using a permutation test on the largest cluster-based statistics^44^ at the significance level of 0.05. This approach allowed for increasing the test sensitivity based on the assumption of temporal continuity of the data, thereby avoiding a massive multiple comparison problem and resulting in continuous intervals. These intervals were calculated separately for gamma and beta burst rates. Second, for each of the resulting intervals, we extracted various burst characteristics, i.e., average number of burst occurrences, their average duration and their average spectral power (over the duration and, respectively, beta or broadband gamma frequency range). Finally, these burst statistics were compared within the intervals using a non-parametric permutation test for exchangeability of condition labels. We also tested whether error trials behaved more similar to either of the correct conditions: within each interval of interest we calculated two statistics: (i) from the difference between burst rates in correct non-match minus incorrect conditions and (ii) difference between burst rates in incorrect minus correct match conditions. Finally, we tested the null hypothesis that the two means were the same. The number of error trials was low, i.e., on average there were 4.7 non-matching and 4.1 matching incorrect trials per session.

### Data availability

All relevant data and code will be available from the corresponding author on reasonable request.

## Electronic supplementary material

Supplementary Information
