# Supplementary material for: Gamma and beta bursts during working memory readout suggest roles in its volitional control
Source: Nat Commun. 2018 Jan 26;9:394. doi: 10.1038/s41467-017-02791-8 (PMC5785952; doi:10.1038/s41467-017-02791-8)
Supplement: Supplementary file 1 — Supplementary Information [file 41467_2017_2791_MOESM1_ESM.pdf]

Supplementary Information

Gamma and beta bursts during working memory read-out suggest roles in its volitional control

Lundqvist et al.

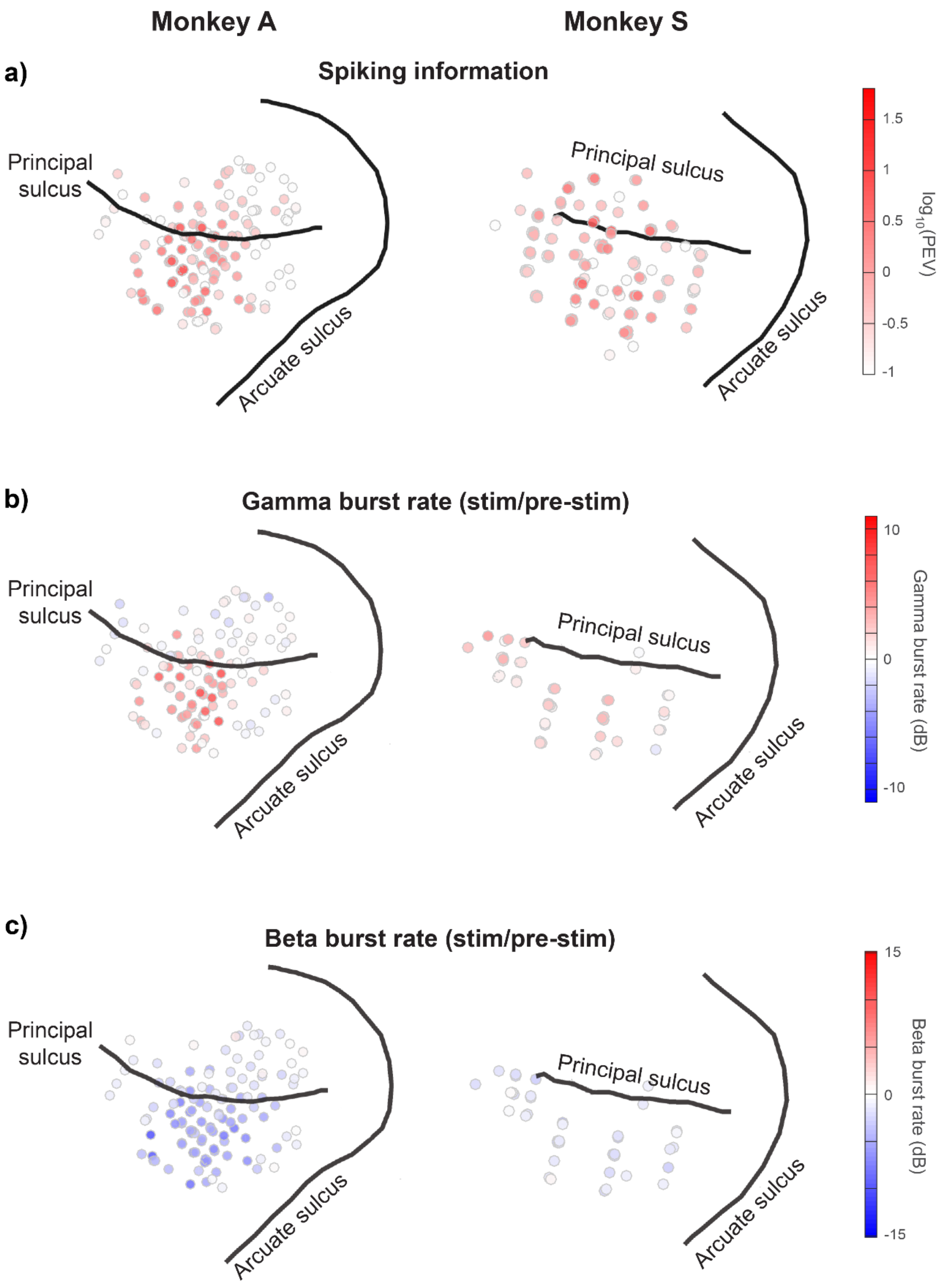

Supplementary Fig. 1. Anatomical distribution of spike information and burst modulation. Distribution of log(PEV), calculated as the maximum spike rate PEV reached by a neuron during the presentation of samples S1 and S2 and the following delays, is shown in a). The anatomical distribution of gamma and beta burst rates induced during the first sample is given in b) and c), respectively. It was calculated as burst rates during first 300 ms of sample, divided by the rates during the 300 ms just preceding sample onset. Induced rates by the second sample, S2, and the first test object, T1, had similar patterns of distribution (not shown).

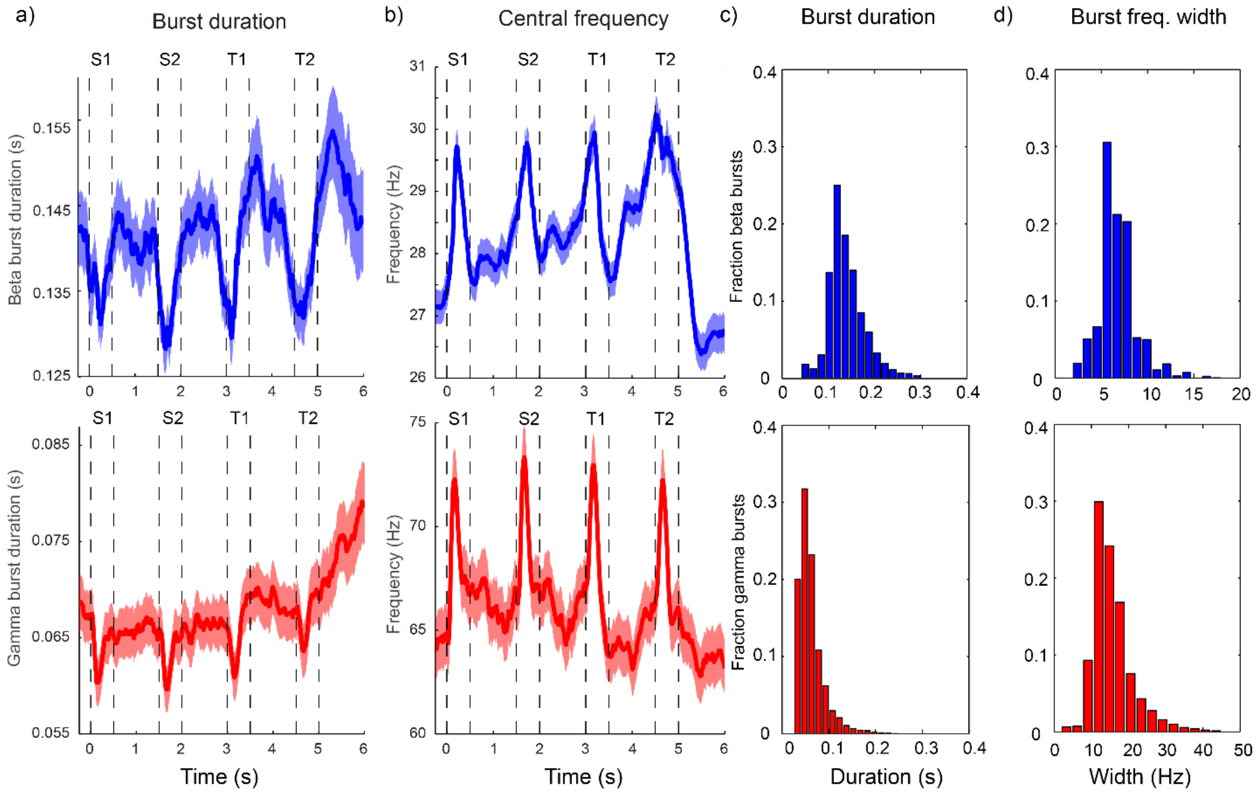

Supplementary Fig. 2. Burst statistics. Top row shows beta (blue), second row shows gamma (red) burst statistics: a) the average burst durations, b) the average central frequency as a function of time, c) the distribution of burst durations, d) the distribution of width of bursts in frequency range. See Methods for details of how these measures were defined. All data from correct match trials from informative electrodes (n=130). Shaded regions represent SEM.

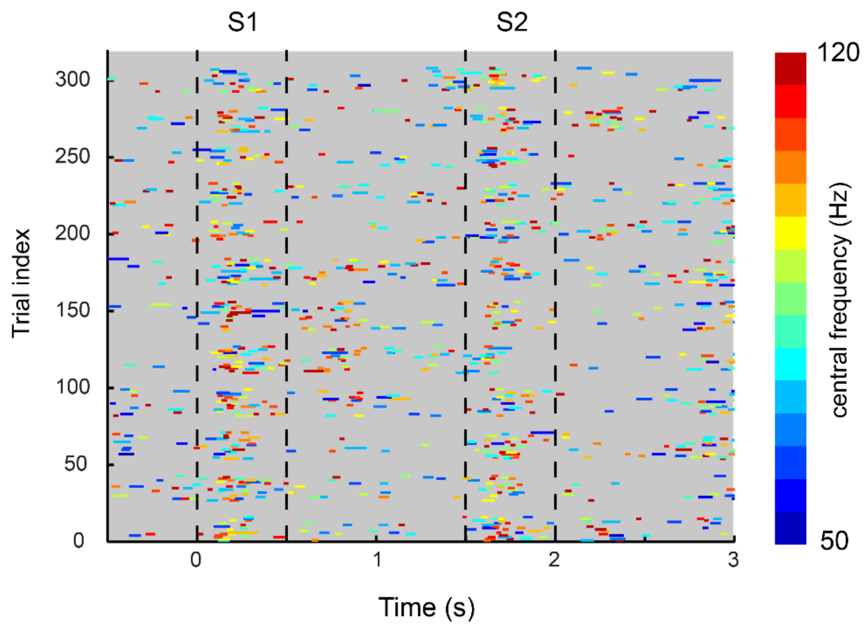

Supplementary Fig. 3. Burst raster plot. Gamma bursts for all correct matching trials during the two samples, S1 and S2, and the following memory delays are shown for the same gamma-modulated electrode as in Fig. 2b, d. Trials are grouped into the 12 different conditions (order and identity for 2 out of 4 objects), with each group separated by a small space. The color code refers to the central frequency of each burst and duration of each burst is shown by extension along the x-axis.

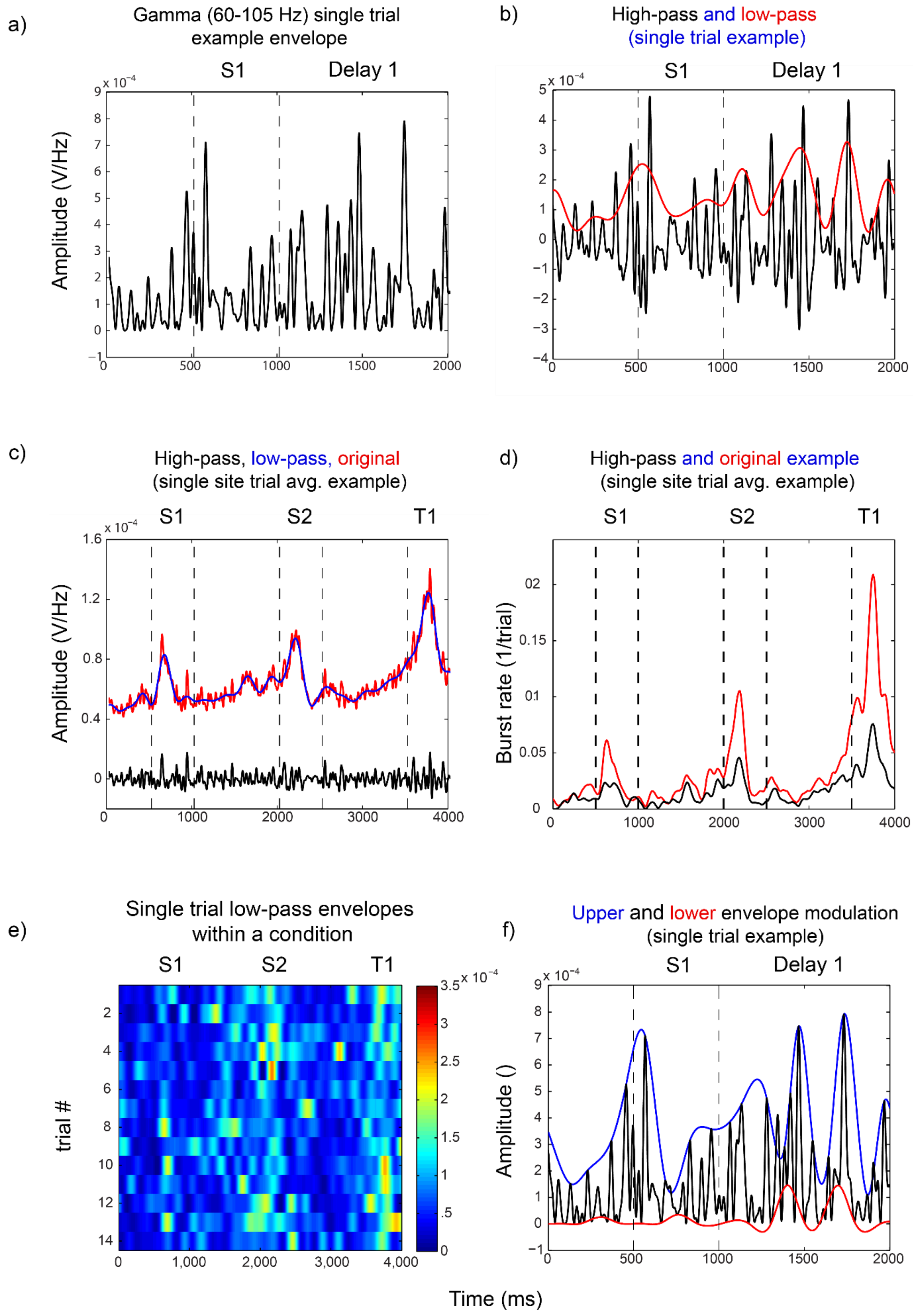
a)
Gamma (60-105 Hz) single trial example envelope
S1
Delay 1
Amplitude (V/Hz)
x 10^-4
b)
High-pass and low-pass (single trial example)
S1
Delay 1
x 10^-4
c)
High-pass, low-pass, original (single site trial avg. example)
S1
S2
T1
Amplitude (V/Hz)
x 10^-4
d)
High-pass and original example (single site trial avg. example)
S1
S2
T1
Burst rate (1/trial)
e)
Single trial low-pass envelopes within a condition
S1
S2
T1
trial #
x 10^-4
f)
Upper and lower envelope modulation (single trial example)
S1
Delay 1
Amplitude ()
x 10^-4
Time (ms)

Supplementary Fig. 4. Non-stationary nature of gamma power. In a) is shown a single trial example of gamma (60-105 Hz) envelope obtained from an electrode displaying gamma-modulation during sample presentations. In b) high-pass (5th order Butterworth filter, 5 Hz; black) and low-pass (5 Hz; red) filtered versions of the same envelope are shown. The trial-averaged gamma amplitude envelope (red) along with trial averages of both the high-pass (black) and low-pass (blue) filtered envelopes are shown in c). In d) is plotted the gamma burst rate estimated based on the high-pass filtered (black) or the original (red) gamma envelopes, corresponding to our canonical approach to burst extraction. All the low-pass filtered gamma envelopes for a specific set of sample and test objects (both order and identity of objects was identical in all shown examples) of the example electrode is shown in e). In f) is shown the same single-trial example as in a), demonstrating how we fit a function to the modulation of the upper (blue) and lower envelope of the signal. These fitted functions were then used to estimate the modulations.

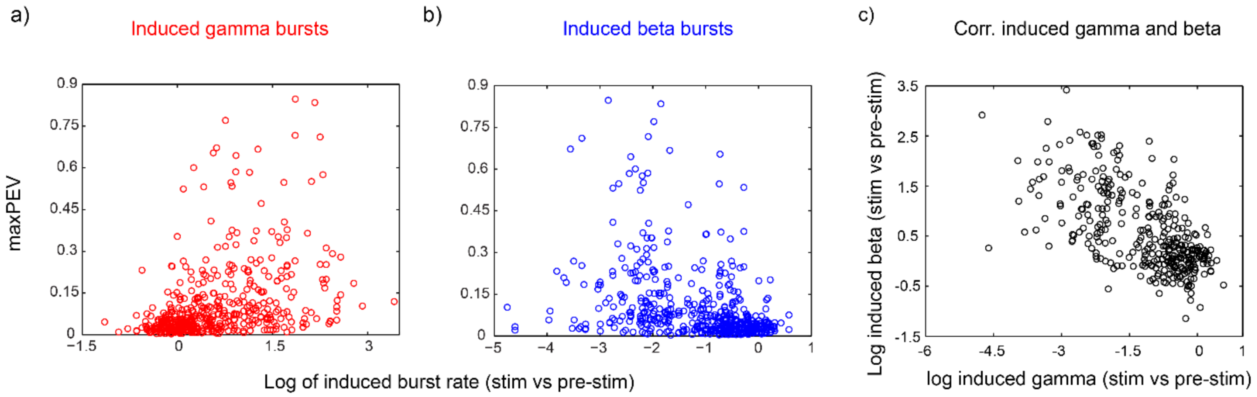

Supplementary Fig. 5. Modulation of burst rates versus spike PEV. a) and b): The abscissa corresponds to the logarithm of the sample induced burst rate. The induced burst rate was defined as the ratio of the burst rate during the first 300 ms of sample presentation and the 300 ms directly preceding sample. The ordinate axis accounts for the maximum PEV (maxPEV) reached in sample or delay epochs for each neuron. In a) is shown induced gamma, and in b) induced beta, VS maxPEV. The correlations between the induced burst rates and maxPEV, reported originally in the main text, were also significantly different from 0 when considering maxPEV separately in sample and delay epochs (maxPEV(sample); Gamma: rho=0.43, p<1.1e-16. Beta: rho= -0.42, p<2e-16. maxPEV(delay); Gamma: rho=0.44, p<1e-16. Beta: rho= -0.39, p<1e-15, number of neurons n=251), Spearman's rank correlation. In c) the log of induced beta is plotted against the log of induced gamma (n=188).

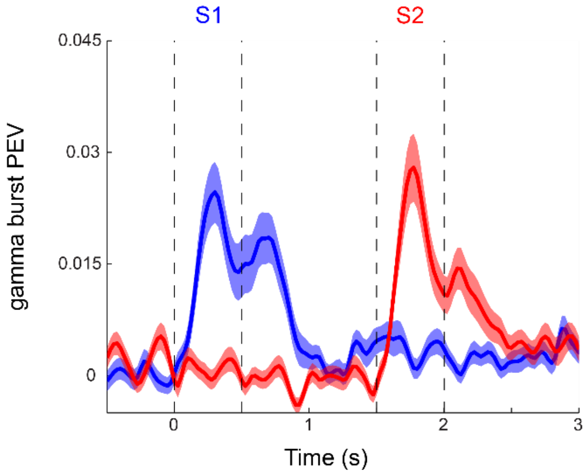

Supplementary Fig. 6. PEV information in gamma bursting. Plotted is the PEV in gamma bursting based on the object identity of sample 1 (blue) and sample 2 (red) for all electrodes with at least one informative unit (n=130). A subset of these showed significant PEVs. In particular, 69/130 of the informative and 5/58 of the non-informative sites had significant PEV in gamma burst rates at some point in the trials ($p<0.05$, corrected for multiple comparisons over time). Shaded regions represent SEM.

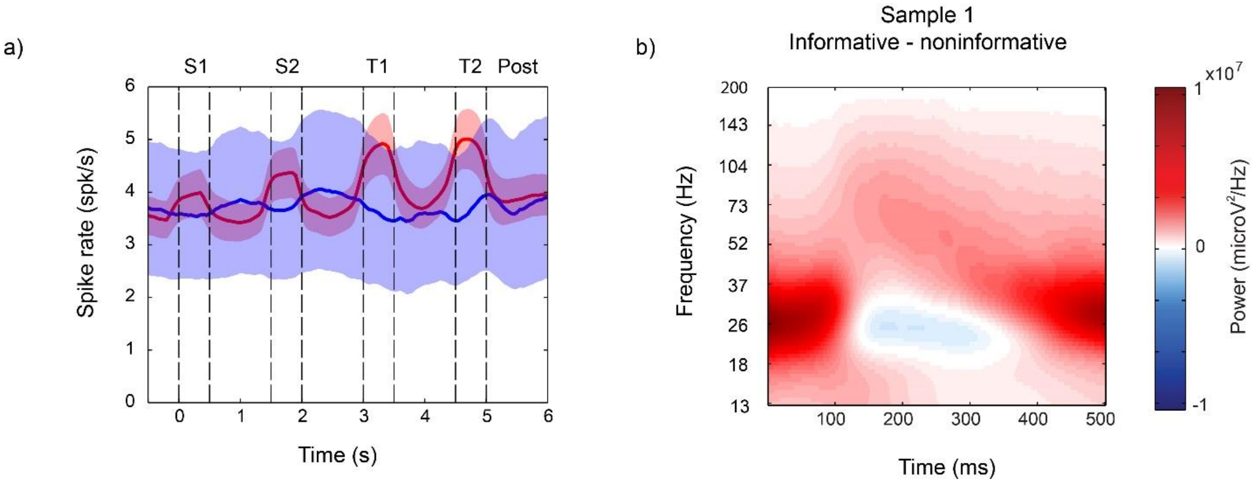

Supplementary Fig. 7. a) Firing rates on gamma-modulated and non-gamma-modulated sites. Plotted in blue are the average firing rates of all neurons (n=39) on sites with no gamma-modulation at stimulus presentations. In red are the average firing rates of all neurons (n=212) on gamma modulated sites. Error bars correspond to SEM. Shown in b) is the difference in spectral power between informative and non-informative sites during the presentation of sample 1, S1.

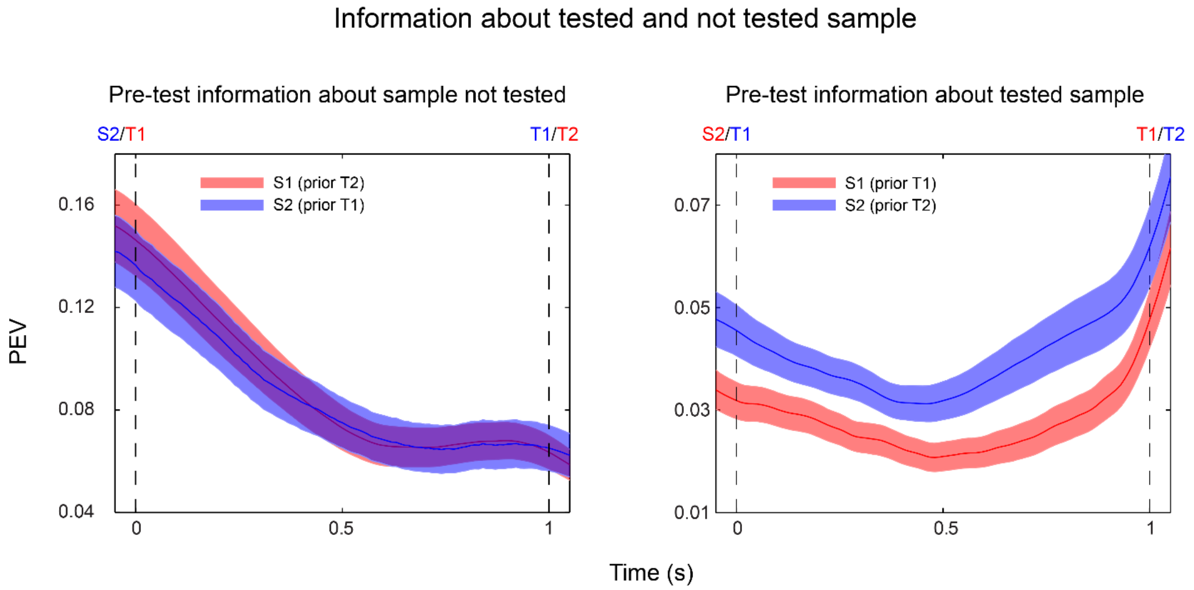

Supplementary Fig. 8. Information about the relevant and non-relevant sample prior to test. To the left is shown the PEV for sample 1 (red) and sample 2 (blue) in the delay leading up to the test of the other sample (two-object delay for sample 2, T1-T2 delay for sample 1). To the right are shown PEVs during the delay of sample 1 (red) and sample 2 (blue) as they are about to be tested (two-object delay for sample 1, T1-T2 delay for sample 2). We used all correct match trials, as there was no S2 ramp up when T1 object did not match S1 object (Fig. 6e). This explains the elevated PEVs in the left plot, as S2 and S1 have just been presented (tested with a matching object for S1, i.e. T1=S1). We used all neurons with delay information in any of the delays (n=186).

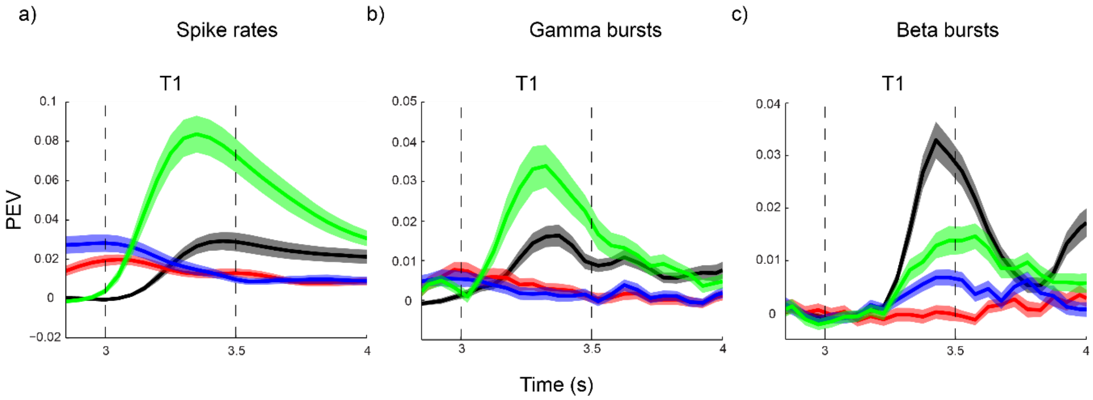

Supplementary Fig. 9. Components of information carried by the neural activity around the first test. Plotted is the PEV about S1 (red), S2 (blue), T1 (green) and the match vs non-match status (black), including data from all correct trials. The PEVs were estimated based on a four-way ANOVA (not including interactions as the matching vs non-matching status would then be confounded by the interaction between sample and test identity). In a) are shown PEVs based on spiking (n=188), in b) based on gamma bursting (n=130) and c) based on beta bursting (n=130) on informative electrodes (note please different axis scaling in the three panels).

Supplementary Note 1

Demonstrating the bursty nature of gamma

We defined bursts as events in which the power envelope for a particular frequency exceeded 2 standard deviations from the mean at a particular electrode (Methods). In single trials the bursts were brief and scattered stochastically across trials (Supplementary Fig. 3). By averaging bursting across trials we arrived at burst rates corresponding to the rate of occurrence of such bursts over time. We interpret these findings as gamma power only being elevated above the baseline in brief scattered bouts and suggest that a weak tendency of bursts to accumulate at particular epochs within a trial gives rise to smoothly varying trial-averaged power. The time-varying burst rates could however conceivably be the result of two distinct dynamics. First, it could indeed be the result of a slowly varying power envelope, similar from trial to trial, but with additive random noise fluctuations. These noise fluctuations would then give the illusion of brief events with high trial variability, whereas the underlying slowly varying power would be similar to the trial-averaged power. Averaging across trials would thus clean the signal from noise and give a better representation of the underlying single trial dynamics. The second option is that there are constant fast transitions between low and brief high power states on single trials. Weak tendencies of high power states to occur at certain epochs in repeated trials gives the illusion of slowly and smoothly varying power dynamics when averaging across trials, but these trends cannot be detected on single trials. The fast 'noise fluctuations' are according to this view a meaningful part of neural dynamics that are lost when averaging across trials. Activity is not as sustained as it appears in the trial average. Here we demonstrate that data appeared to support the second model.

In Supplementary Fig. 4a a representative single-trial, single-recording-site example of a gamma power envelope (60-105 Hz) is shown. It made fast transitions from a baseline level close to 0 to high power states throughout the trial. The transitions occurred both during fixation, sample presentation and the following delay. Supplementary Fig. 4b shows the same signal high-pass (black, 5 Hz) and low-pass (red, 5 Hz) filtered using 5th order Butterworth filters. The low-pass filtered single-trial envelopes contained all the contributions to the trial averaged power (Supplementary Fig. 4c), whereas the high-pass filtered envelopes averaged to 0 since the slow trends have been removed. To demonstrate that our trial-averaged burst rates did not depend on slow trends we extracted bursts from these high-pass filtered envelopes. This yielded burst rates that were highly correlated with the burst rates extracted from the original envelope (Supplementary Fig. 4d, mean correlation 0.69, s.d 0.11 calculated from 500 ms before the first sample up until the first test stimulus presentation using all informative electrodes, n=130). The burst rates could thus be recovered from the fluctuations alone. At a single-trial level the fluctuations (bursts) tended to occur at the same time as positive contributions to the trial-averaged power from the low-pass filtered envelope (Supplementary Fig. 4b). This could be demonstrated by correlating over time the variance (running average, 100 ms time window) of the high-pass filtered signal with the amplitude of the low-passed signal for each trial and recording site (mean correlation 0.62, s. d. 0.02, n=130). In other words, at a single-trial level the fluctuations (bursts) and positive contributions to the trial-averaged power tended to coincide in time.

Further, the slow trend (the low-passed envelope) exhibited high trial-to-trial variability (Supplementary Fig. 4e). This was estimated by correlating all pairs of single trial low-pass envelopes sharing the same condition (the same identity and order of sample and test objects), and then averaging across conditions (mean correlation 0.08, s.d. 0.07). Thus, in sum, there tended to be more bursts during periods of single trials with a positive contribution to the trial-averaged power. However, such epochs were only weakly correlated across trials (this weak correlation in turn gave rise to the changes over time in the trial average). This is consistent with the view that transient and highly variable dynamics underlie trends in trial-averaged data.

Still, these findings could conceivably originate from two distinct mechanisms. 1) Increased fluctuations in gamma band power, reflected in detected bursts, could be driven by the underlying trends in the average spectral power (if the variance of band power scaled with the mean, change in the mean would drive fluctuations). 2) Alternatively, brief episodes of spectral band power increase (positive deflections), measured as bursts, could instead contribute to shaping the slow single-trial trends (with a series of bursts accompanying a positive contribution to power). The key distinction between the two mechanisms lies in the nature of the power fluctuations: they could be symmetric around a mean (as in the high-pass filtered envelope example; Supplementary Fig. 4b) that varies on a time-scale slower than the bursts themselves, or manifest themselves as positive deflections from a baseline level stable throughout the trial (as in the original gamma envelope example, Fig S4a). To differentiate between these alternative explanations we examined the modulation of the lower (red) and upper (black) envelope of each unfiltered gamma envelope (Fig S4f). The upper and lower components were obtained from the gamma envelope by a spline approximation (with smoothing of 80ms) of the curve connecting its peaks and troughs. We then separately estimated the modulation of the lower and upper components for each trial by evaluating the maximal amplitude (among the differences between peaks and troughs of the floor and ceiling signals, respectively) within 0.5-s-long moving windows (local neighborhood for peaks and troughs). The largest amplitude was selected to represent each trial. Longer windows (0.5-1.0s) had no qualitative effect on the outcomes. If the power signal was dominated by positive deflections from a flat baseline (rather than fluctuations in both directions scaling with a slowly changing mean), the upper envelope should be modulated much more strongly than the lower envelope. Indeed, the modulation of the upper component of the gamma envelope was on average (across the trials and informative electrodes) 7.8 times larger than that of the lower component.

Information in gamma burst rates

According to our model, gamma bursting is associated with informative spiking of cell assemblies but itself it carries little information. Any information accounted for gamma burst occurrences is a by-product of non-overlapping parts of the assemblies. Consistently with this hypothesis, we found a subset of the sites where gamma burst rates were informative of object identity (see Supplementary Fig. 7 and Supplementary Fig. 9). The overall pattern of spike rate and gamma burst rate PEVs were very similar, but consistently lower for gamma burst rates. On the sites with significant burst rate PEV (69/130), it was highly correlated with the spike rate PEV of the local neurons (avg. $r=0.45$, $p<1e-9$, Student's t-test for non-zero correlation following Fisher's z-transformation, $n=69$). To obtain more insights into the relationship between the information accounted for by spiking and gamma bursting, i.e. to examine whether spiking and gamma bursting were co-modulated with respect to the same preferred conditions thus

explaining the same variance sources, we conducted a separate two-way ANOVA test for interactions. One factor corresponded to the identity of the object presented in S1 and the other factor was constituted by a categorical variable describing the type of neural activity, spiking or gamma bursting, used as the dependent measure in the analysis. The two neural activity measures were averaged over the first presentation interval (S1) and to ensure that interaction effects were not due to their baseline differences, we normalized them to have zero mean and unity variance across the trials. We only included sites where both the neuron and gamma bursting had significant PEVs ($p<0.05$) in two one-way ANOVA tests) during presentation of the first object (n=71). With spikes and gamma bursts combined, there was the main PEV effect of object identity (mean $\omega^2=0.08$), suggesting that both neural measures tended to prefer the same objects. However, out of the aforementioned 71 neurons, which had significant PEV in both spiking and gamma bursting, most (76% or 54/71) showed also a significant interaction effect between the measure type and object identity (mean effect size $\omega^2=0.03$). This indicates that they did not carry the exact same information, and there were some discrepancies in their object preferences.

In conclusion, the PEV information carried by spiking and gamma bursting was correlated over time with a tendency to peak in the same time intervals on single sites. It also tended to be similar, but not identical, with regards to preferred category, suggesting gamma and spiking were independent measures. This is consistent with our model and conclusions from other analyses. We view the elevated gamma bursting, representing population activity, as a condition/prerequisite for elevated spiking of single neurons. Gamma bursts reflect the activation of a nearby cell assembly, whereas individual neurons belong to a subset of these spatially overlapping assemblies. The neurons then display elevated firing rates in a subset of such occasions of elevated gamma, hence displaying higher PEV by object identity, at times when gamma burst rates (and PEVs) are elevated. The significant interaction between measure (spike, burst) and object identity indeed suggests that individual neurons do not strictly follow local burst rates.

Single-trial relation between spiking, information and oscillatory bursts

Information measured by PEV (Methods) is estimated across trials. The tested model predicts that when the network is producing a burst of gamma it is in a state with increased firing rates as well as spiking information. To test this prediction we used the burst times as a mask for filtering the spike rates on a trial-by-trial basis. We then calculated PEVs across trials based on these spike rates estimated during gamma bursts only. To estimate if spiking was more informative during gamma bursts compared to other times with the same distribution, we shuffled the trial identities for burst times relative spike times. We then recalculated PEVs for each of 1,000 shuffles. The shuffling was performed only within identical conditions (the same items being presented) to account for correlated changes in spike and burst rates with condition. We focused this investigation on the first delay interval (S1-S2), when only one item had been presented and there was no ramp-up/time varying patterns in gamma to minimize the effect of undesirable sources of variability.

The spike rate during the first delay was significantly higher in gamma bursts (spike rate for original burst location: 5.32, shuffled: 4.20 spikes/s, $p<0.0001$, n=146, Wilcoxon rank test). Gamma bursts were also associated with higher spike PEV by grouping trials by presented

object (PEV for original burst location: 0.058, shuffled: 0.047, p=0.02, n=146, Wilcoxon rank test). To ensure that the increased PEV was not due to an overall decrease in variance inside gamma bursts we estimated total variance in the original and shuffled masked spike trains. The variance was however higher inside the gamma bursts (var. spike rate for original burst location: 53.9, shuffled: 43.2, p<0.0001, n=146, Wilcoxon rank test). Thus, spike rates, their variance as well as the amount of variance that could be explained by maintained object identity were significantly higher during gamma bursts.

We tested the prediction that beta was associated with the suppression of information and spiking in an analogous way. This analysis showed that spike rates were reduced in beta bursts (spike rate for original burst location: 3.71, shuffled: 4.06 spikes/s; p=0.004, n=146; Wilcoxon rank test). The total variance was also reduced in the beta bursts (var. spike rate for original burst location: 38.9, shuffled: 44.4, p=0.05, n=146, Wilcoxon rank test), whereas spike PEV was reduced but not significantly (PEV for original burst location: 0.043, shuffled: 0.045, p=0.14, n=146, Wilcoxon rank test). We repeated this analysis using actual spike counts rather than estimated spike rates inside bursts with qualitatively identical results (not shown).

	Beta Interval 1	Beta interval 2	Gamma interval 1	Gamma interval 2	Gamma interval 3
Corr. Match Vs. Corr. 1st Nonmatch.	p=0.12 n=130	p=0.31 n=130	p=0.70 n=130	p=0.09 n=130	**p=0.04*** n=130
Corr. Match Vs. Incorr. Match	p=0.55 n=117	p=0.88 n=117	p=0.23 n=117	p=0.94 n=117	p=0.37 n=117
Corr. Match Vs. Incorr. 1st Nonmatch	p=0.11 n=118	p=0.14 n=118	p=0.66 n=118	p=0.72 n=118	p=0.16 n=118
Corr. 1st Nonmatch vs Incorr. Match	p=0.88 n=117	p=0.63 n=117	p=0.14 n=117	p=0.37 n=117	p=0.65 n=117
Corr. 1st Nonm vs Incorr. 1st Nonmatch	p=0.71 n=118	p=0.17 n=118	p=0.41 n=118	p=0.45 n=118	**p=0.02*** n=118

Supplementary Table 1. Power inside bursts. For the intervals defined by differences in burst rate (three intervals in gamma, two in beta, columns; see Figure 7) we tested significant differences in average power inside bursts between the conditions (5 pairs in rows). Statistics were calculated using two-sided permutation tests (10000 permutations).
